# Inference-based accuracy of metagenome prediction tools varies across sample types and functional categories

**DOI:** 10.1186/s40168-020-00815-y

**Published:** 2020-04-02

**Authors:** Shan Sun, Roshonda B. Jones, Anthony A. Fodor

**Affiliations:** 1 Department of Bioinformatics and Genomics, University of North Carolina at Charlotte, Charlotte, NC USA; 2 The Saban Research Institute, Children’s Hospital Los Angeles, University of Southern California, Los Angeles, CA USA

**Keywords:** Microbiota functional profile prediction, Inference, Sample type, Functional category

## Abstract

**Background:**

Despite recent decreases in the cost of sequencing, shotgun metagenome sequencing remains more expensive compared with 16S rRNA amplicon sequencing. Methods have been developed to predict the functional profiles of microbial communities based on their taxonomic composition. In this study, we evaluated the performance of three commonly used metagenome prediction tools (PICRUSt, PICRUSt2, and Tax4Fun) by comparing the significance of the differential abundance of predicted functional gene profiles to those from shotgun metagenome sequencing across different environments.

**Results:**

We selected 7 datasets of human, non-human animal, and environmental (soil) samples that have publicly available 16S rRNA and shotgun metagenome sequences. As we would expect based on previous literature, strong Spearman correlations were observed between predicted gene compositions and gene relative abundance measured with shotgun metagenome sequencing. However, these strong correlations were preserved even when the abundance of genes were permuted across samples. This suggests that simple correlation coefficient is a highly unreliable measure for the performance of metagenome prediction tools. As an alternative, we compared the performance of genes predicted with PICRUSt, PICRUSt2, and Tax4Fun to sequenced metagenome genes in inference models associated with metadata within each dataset. With this approach, we found reasonable performance for human datasets, with the metagenome prediction tools performing better for inference on genes related to “housekeeping” functions. However, their performance degraded sharply outside of human datasets when used for inference.

**Conclusion:**

We conclude that the utility of PICRUSt, PICRUSt2, and Tax4Fun for inference with the default database is likely limited outside of human samples and that development of tools for gene prediction specific to different non-human and environmental samples is warranted.

Video abstract.

## Introduction

Recent advances in next generation sequencing are revolutionizing our understanding of complex microbial communities. Amplicon sequencing of marker genes provides information regarding the phylogenetic diversity and taxonomic composition of microorganisms present in the environment, while shotgun metagenome sequencing provides additional information on the relative abundance of functional genes. Although knowledge of taxonomy and functional genes of microorganisms are both important, functional genes are more directly related to pathways and therefore are essential for understanding the roles microorganisms play with regard to different physiological or ecological outcomes. However, the higher cost of metagenome sequencing hinders its application in studies consisting of a large number of samples, which are usually necessary in order to ensure adequate statistical power for detecting true differences [1]. Additionally, metagenome sequencing can also be very challenging for low biomass samples or samples that are dominated by non-microbial DNA [2, 3].

To address this problem, tools have been developed to predict microbial functional genes from their taxonomic compositions inferred from more cost-effective amplicon sequencing, including PICRUSt, PICRUSt2, Tax4Fun, and FaproTax [4–7], and these tools have been applied in hundreds of projects on various environments, including human gut [8, 9], murine [10, 11], fish [12], coral [13], water [14], plant [15], bioreactor [16], and soil [17]*.* The algorithms generally predict the genes of organisms without sequenced genomes based on mapping their 16S rRNA genes to homologous taxa with fully sequenced genomes. Thus, the predictions are limited by currently available genomes, which are highly biased towards microorganisms associated with human health and biotechnology use [18].

To gauge the reliability of the predictions of these tools in different environments and for different functional categories, we utilized human, non-human animal (gorilla, mouse, and chicken), and environmental (soil) datasets that were sequenced for both 16S rRNA marker genes and shotgun metagenomes. We compared the predicted functional profiles to the functional profiles measured with shotgun metagenome sequencing. We demonstrated that simple correlations such as Spearman correlation overstate the accuracy of the metagenome prediction tools by not taking into account the low variance of functional profiles generated from shotgun metagenome sequencing. As an alternative metric, we used the predicted results for inference with simple statistical models and found reasonable performance for human datasets, which presumably reflected the better reference information we currently have for human genomes, but a sharp decrease in performance for inference in non-human samples. The evaluation of metagenome prediction tools’ performance also indicated that the accuracy of prediction varies by functional categories with typically better performance for genes related to “housekeeping” functions, possibly due to the difficulty predicting genes with higher phylogenetic variability, higher horizontal gene transfer rates, or genes related to the unculturable state of the microorganism. The variable performance across environments and functional categories should be considered when interpreting the results of metagenome prediction tools.

## Results

### Spearman correlation is not a reliable measurement for the prediction accuracy of gene contents

**Fig. 1:**
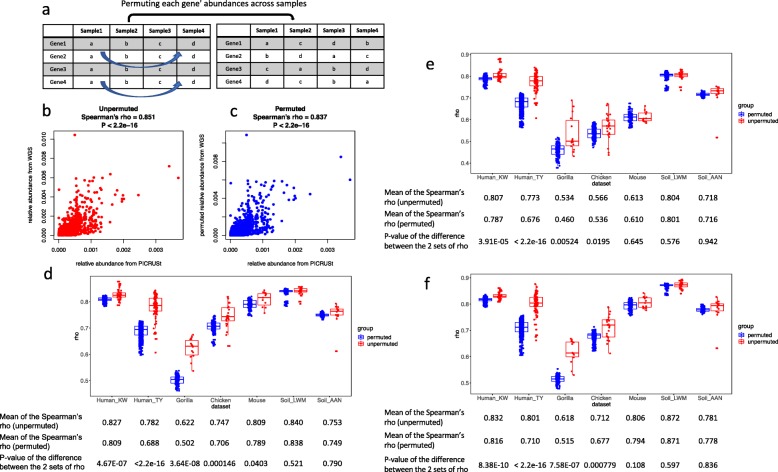
Spearman correlations between metagenome predictions and shotgun metagenome sequencing in unpermuted and permuted datasets. **a** Each gene’s abundance was permuted across samples independently. **b** and **c** An example showing the correlations between genes relative abundances estimated by PICRUSt and metagenome sequencing in a soil sample (sample BulkAG3 in soil_AAN dataset) for unpermuted (**b**) and permuted (**c**) data. **d–f** The Spearman correlations of gene composition estimated from metagenome sequencing and predicted with PICRUSt (**d**), PICRUSt2 (**e**), and Tax4Fun (**f**) in unpermuted (red) and permuted data (blue) in all datasets. In each of the 100 permutations, every gene’s abundance was permuted across samples independently

We compared the predictions of PICRUSt, PICRUSt2, and Tax4Fun to the results of shotgun metagenome sequencing on publicly available datasets for which both metagenome and 16S rRNA sequences were available (Table S1). As we would expect from previous literature [4], gene content estimations from these tools were robustly correlated with gene contents from metagenome sequencing with Spearman correlations in the range of 0.53 to 0.87 (Fig. 1). For example, in one soil sample (Fig. 1b), there is a clear correlation between the relative abundance of each gene from PICRUSt and the relative abundance from metagenome sequencing (Spearman’s rho = 0.85). However, if we independently permute each gene’s abundances across samples (Fig. 1a) and then compare the gene composition from metagenome sequencing to PICRUSt predictions of this sample, the correlation that was observed is not substantially impacted (Spearman’s rho = 0.84) (Fig. 1c).

**Fig. 2:**
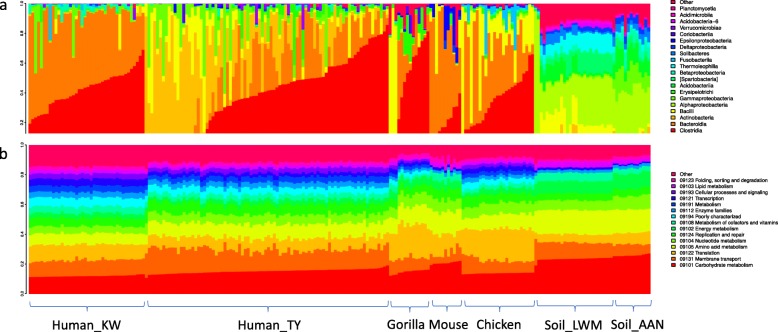
Taxonomic (**a**) and functional profiles (**b**) of the 7 datasets in our study. The taxonomic profiles were plotted at the class level, and the functional profiles were plotted at the broadest functional category of the KEGG database for visualization

The likely explanation for this observation is that across environments, there is less variation between metagenome functional profiles of samples than their taxonomic profiles (Fig. 2), an observation that has been previously made for human samples in the Human Microbiome Project [19]. In the datasets examined, the relative abundance of genes from prediction tool estimates were highly correlated with that from metagenome sequencing, with correlation coefficients always higher than 0.5, and this was true for both permuted and unpermuted samples for PICRUSt (Fig. 1d), PICRUSt2 (Fig. 1e), and Tax4Fun (Fig. 1f). The correlations were often only marginally higher on the unpermuted data than those permuted, with perhaps the gorilla dataset as an exception (Fig. 1d, e, and f). However, even in the gorilla samples, the largest difference between Spearman coefficients for permuted and unpermuted data was only 0.12. For the 2 soil datasets, the Spearman coefficients for the unpermuted data were not significantly different from those for the permuted ones with all three prediction tools (Fig. 1d, e, and f).

### Inference from metagenome prediction tools showed higher consistency with metagenome sequencing in human samples than non-human samples

**Fig. 3:**
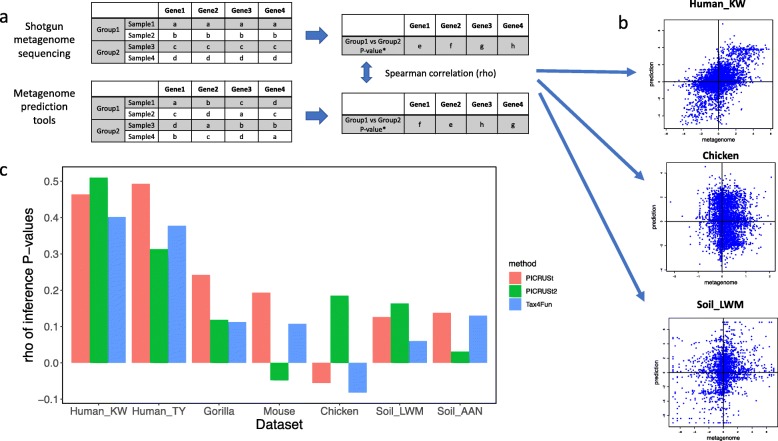
Comparison of inferences based on gene composition estimated with metagenome prediction tools and metagenome sequencing in each of the 7 datasets. **a** In this approach, *P* values of the Wilcoxon test evaluating the null hypothesis for each dataset (see the “Methods” section and Table S1) were calculated for metagenome sequencing and metagenome prediction tools. The *P* values for genes in common between the two methods were compared using Spearman’s correlation, and the resulting rho was considered as an estimate for the correlation of inference. **b** Examples showing the correlations between the *P* values from metagenome prediction tools and metagenome sequencing in Human_KW, chicken, and Soil_LWM datasets. For example, in the Human_KW dataset, genes higher in urban subjects are in the upper-right hand quadrant, and genes lower in urban are in the lower-left hand quadrant. **c** The correlation of inference between metagenome sequencing and PICRUSt, PICRUSt2, and Tax4Fun for all seven datasets

**Fig. 4:**
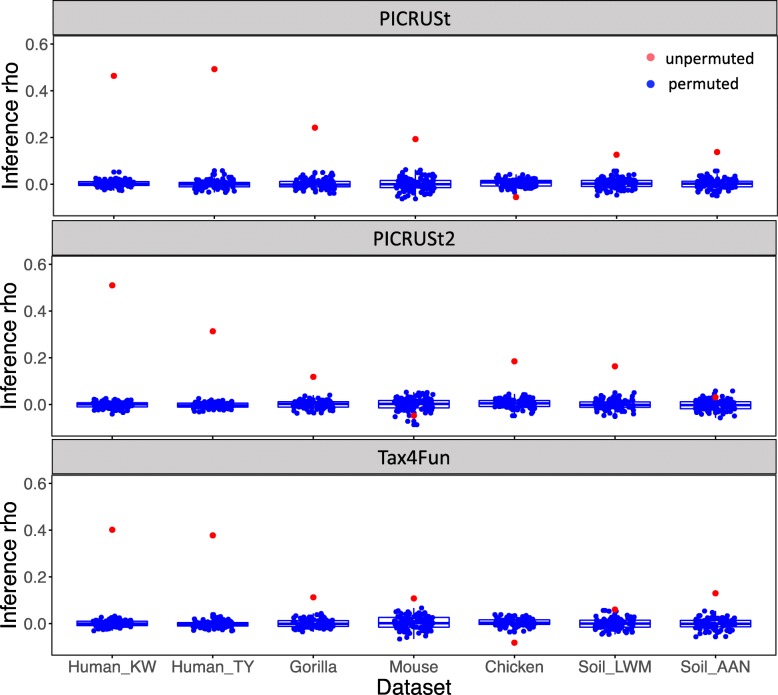
The results of inference methods in unpermuted and permuted datasets. The red points are the inference correlations between metagenome prediction tools and unpermuted metagenome sequencing data for each dataset. The boxplots of blue points show the inference correlations between metagenome prediction tools and permuted metagenome sequencing data for 100 permutations. In each of the 100 permutations, every gene’s abundance was permuted across samples independently

As an alternative evaluation to Spearman’s correlation of gene composition, we examined how the inference of predicted gene compositions compared to that of shotgun metagenome sequencing in each of our datasets. For this purpose, we formed a null hypothesis for each gene in each dataset that there is no difference in the mean of that gene’s distribution of relative abundance between the two groups in the dataset. For example, for each of the 5574 genes detected by both PICRUSt and metagenome sequencing in the Human_KW dataset, we used a Wilcoxon test to generate *P* values for the difference in gene composition between rural and urban samples. Across all the genes, there was a reasonable correlation (rho = 0.46) of *P* values from Wilcoxon tests run on real metagenome sequencing data and those predicted with PICRUSt data. Unlike our results for Spearman correlation of gene composition, this inference correlation is sensitive to data permutation, as when we repeated this procedure on permuted data (Fig. 3a), the correlation between *P* values generated from metagenome sequencing and those from prediction tools approached zero (Fig. 4). We calculated the inference correlation coefficients for the estimates from PICRUSt, PICRUSt2, and Tax4Fun on all 7 datasets. We saw a similarly robust correlation for the other human dataset (Human_TY) evaluating a null hypothesis comparing the US and non-US samples. However, when we extended this analysis to non-human datasets (using the null hypotheses for each study listed in Table S1), the inference produced by metagenome prediction tools showed a much lower similarity to inference produced by metagenome sequencing (Fig. 3c).

To determine whether sample sizes contributed to the differences in performance across datasets, we randomly subsampled each larger dataset (without replacement) to 10 samples (5 per group) and re-calculated the comparison of *P* values between metagenome prediction tools and metagenome sequencing (Fig. S1). Even at a smaller size, data from the human studies showed greater concordance than those from other environments. We conclude that the difference in sample sizes between datasets does not explain the variability of metagenome prediction tools’ accuracy between different sample types in our study. Likewise, the effect sizes of the associations with metadata, measured as *R*^2^ in a PERMANOVA test, were not substantially higher in human samples (Table S1). It therefore also seems unlikely that effect size alone can explain the better concordance we observed between inference results from metagenome prediction tools and metagenome sequencing for human samples.

We further investigated the consistency of metagenome prediction tools and metagenome sequencing by examining how many genes were missed or incorrectly detected by metagenome prediction tools. For some datasets, such as the Human_KW dataset, metagenome prediction tools failed to predict many genes that were detected by metagenome sequencing (Table S2). For other datasets, such as the soil datasets, many genes predicted were not detected in metagenome sequencing, and there were also many genes seen in metagenome sequencing but not in metagenome prediction tools (Table S2). For the chicken dataset with an average metagenome sequencing depth of 31 million reads/sample and the gorilla dataset of 27 million reads/sample, 39.5% and 36.9% of predicted genes could not be detected by metagenome sequencing. In addition, the metagenome sequencing of the Human_KW dataset with an average sequencing depth of 10 million reads/sample detected 13,880 genes and metagenome prediction tools missed 59.1% of them.

### Metagenome prediction tools performs differently for different functional categories

**Fig. 5:**
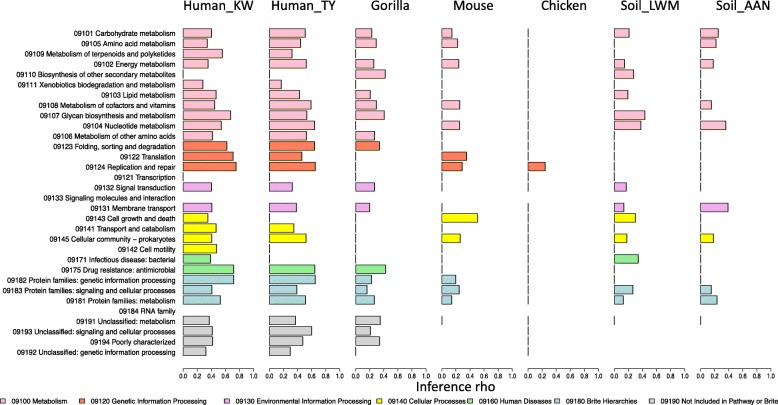
Inference correlations between PICRUSt and metagenome sequencing in 32 KEGG functional categories at the second hierarchy level with the bar colors indicating the functional categories at the first hierarchy level. The results of PICRUSt2 and Tax4Fun are shown in Fig. S2. Negative and insignificant correlations are not shown

We next investigated the discrepancy between metagenome prediction tools and metagenome sequencing for inference in different functional categories (Fig. 5, Fig. S2-4). When comparing the inference from metagenome prediction tools to inference from metagenome sequencing, some functional categories performed better than others in the human gut samples, including those related with genetic information processing such as replication and repair, translation, folding, sorting and degradation, and metabolism-related functions including glycan biosynthesis and metabolism, nucleotide metabolism, and amino acid metabolism. Some functional categories performed less well, including biosynthesis of other secondary metabolites, xenobiotics biodegradation and metabolism, and functions related with environmental information processing and signaling and cellular processes, such as signal transduction, membrane transport, and cell growth and death. For the genes only detected by one method, most of the genes missed by metagenome prediction tools belong to signal transduction, signaling molecules and interaction, and functions related with genetic information processing, while metabolism-related functions were more likely to be predicted (Fig. S3 and Table S3c). Among the genes predicted by metagenome prediction tools but not detected by metagenome sequencing, most of them belong to signaling molecules and interaction, metabolism of terpenoids and polyketides, and xenobiotics biodegradation and metabolism (Fig. S4 and Table S3d).

## Discussion

Microbial community functional profiles are typically of much lower variance compared to their taxonomic profiles [19–21], likely because of the large proportions of “core” or “housekeeping” functions. Likewise, specific niche adaption pathways may contribute to overall microbial functional stability in specific environments [22]. In this study, we showed that this lack of variance in functional profiles between samples leads to a strong correlation between functional profiles from metagenome sequencing and those estimated from references with metagenome prediction tools, even when the gene compositions are permuted across samples (Fig. 1d–f). Because of the relative functional stability of the microbiota in certain environments, metagenome prediction tools could likely better predict average gene profiles rather than within-niche variations. We argue that this result shows that metrics commonly used to measure gene prediction performance, such as Spearman correlation between gene composition estimated with prediction tools and metagenome sequencing, do not give a satisfactory measure of overall accuracy. As an alternative, we evaluated the performance of three commonly used metagenome prediction tools at a community level based on inference from simple statistical models testing the association between genes and metadata. Unlike simple Spearman correlations of gene compositions, evaluation with inference methods is highly sensitive to data permutation (Fig. 4), which indicated that inference methods are much less affected by the relatively low variance of functional profiles. The inference-based approach also has the advantage of reflecting the common use of metagenome prediction tools to reveal predicted functional profiles associated with different metadata categories [12, 13, 23–25]. Incorrect estimation of differential abundance could lead to false discovery of signature genes, and this concern motivated our approach to determine the reliability of inference produced with metagenome prediction tools in different ecosystems.

In this study, we selected 7 datasets from different environments which include human, non-human animal, and environmental (soil) samples. With inference methods, we found that metagenome prediction tools and metagenome sequencing had more consistent assessment from human datasets than non-human animals or environmental datasets. It is likely that these differences reflect the bias of genome databases towards human-related microorganisms. However, metagenome prediction tools still missed a large percentage of genes that were detected with metagenome sequencing in human samples, and an increase in metagenome sequencing depth could presumably increase the number of genes that are potentially not detected by metagenome prediction tools (Table S2). Likewise, metagenome prediction tools sometimes predicted many genes not found in metagenome sequencing even in samples with presumably adequate sequencing depth of millions of reads per sample, which suggested that these additional genes are likely incorrect predictions (Table S2). Discordance between databases used for gene prediction tools and KEGG pathways, which are frequently updated, or other issues in ontology or annotation systems that differ between methods could also contribute to the lack of common gene nomenclature between shotgun metagenome sequencing data and prediction tools.

As a meta-analysis across multiple studies, there are systemic factors that may influence the results of this study, including different sample sizes, sequencing designs, and effect sizes of associations with the metadata. We repeated our analysis on subsampled datasets that were rarified to the number of samples in the smallest dataset that we examined and observed a similar pattern of results with inference more consistent between metagenome prediction tools and metagenome sequencing for human studies (Fig. S1). This result suggests that difference in sample size does not explain the better inference performance for the human studies. While differences in effect size and experimental design are harder to control, the human studies did not have an obviously higher effect size than the non-human studies as measured with a PERMANOVA test (Table S1). It therefore also seems unlikely that differences in effect sizes of associations with the metadata can explain our results.

Our study also examined the performance of metagenome prediction tools for different functional categories. This approach was motivated by the presumed bias in current genome databases toward culturable microorganisms [26]. We reasoned that the unculturable state of microorganisms could be caused by specific requirements for nutrients, temperature, pH, beneficial interactions with other microbes, or extremely slow growth rates [27], which in turn could lead to bias in gene families in different microorganisms. Likewise, different microorganisms and genes also have different rates of horizontal gene transfer, and the accuracy of gene content estimation may therefore vary depending on the type of the genes and microbial groups [28]. We found that metagenome prediction tools generally performed best for “housekeeping” functions such as those related with genetic information processing while the accuracy of functions related to environmental information processing, secondary metabolites, and xenobiotics metabolism was generally much lower (Fig. 5), possibly because the low phylogenetic variability of genes involved in core functions leads to more accurate prediction. Future algorithms for gene prediction could explicitly incorporate this performance variance into a confidence score that could give users estimated error rates for prediction of a given gene family.

In comparing the three methods, we evaluated (PICRUSt, PICRUSt2, and Tax4Fun); no method was obviously superior to another. The prediction of some methods had a higher correlation to the metagenome sequencing data on particular samples, such as PICRUSt2 on the chicken dataset, but PICRUSt2 performed less well in capturing the inference pattern from real metagenome sequences in some other datasets such as mouse. Overall, our results do not support a baseline recommendation of one of these methods over the others.

Our analysis suggests that in order to better predict microbial functional profiles in certain environments, it will be of utility to develop tools specific to that environment. There have been encouraging examples in the literature of efforts to make environmental specific databases such as CowPI, a functional inference tool specific to the rumen microbiome, which had better estimates than PICRUSt when used for predicting functional profiles in the bovine environment [29]. We can look forward to similar future refinements in the next generation of these algorithms that will use appropriate reference databases for an environment and analyze individual functional categories to yield confidence scores for each prediction.

## Conclusions

Our analysis argues that the low variance of microbial functional profiles makes Spearman correlation of gene composition an unreliable metric for evaluating the accuracy of predicted functional gene profiles from taxonomic profiles. As an alternative to simple correlations, we utilized an inference-based method and found poor agreement between metagenome prediction tools and metagenome sequencing outside of human samples and housekeeping genes. This suggests the necessity of future tool development specific to non-human environments that explicitly considers gene functional category as part of the model building process.

## Methods

The datasets used in this study include 2 human datasets (named as Human_KW [30] and Human_TY [31] in our study after the initials of their first authors), 1 gorilla [32], 1 mouse [33], 1 chicken [34], and 2 soil datasets Soil_LWM [35] and Soil_AAN [36]. Each dataset has publicly available 16S rRNA and metagenome sequences and is associated with a two-level categorical metadata. The Human_KW study compared urban and rural subjects in China, while the US and non-US subjects were compared for the Human_TY study. In the gorilla study, the dry and wet seasons were compared while the mouse study compared community composition of two enterotypes. Lean and fat broiler chicken lines were compared for the chicken study. For the Soil_LWM study, Amazon dark earth and agricultural soil were compared, while forested and deforested soils were compared for the Soil_AAN study. Information regarding data locations, sequencing depth, sample sizes, and effect sizes (measured as *R*^2^ in the PERMANOVA test with the function “adonis” in the R package “vegan”) for each study are listed in Table S1.

The PICRUSt, PICRUSt2, and Tax4Fun predictions of the 16S rRNA sequences in the datasets followed the developer’s instructions [4, 5, 7]. The authors’ metagenome analysis results were used when available [31, 33, 35, 36]; otherwise, the raw sequences were analyzed with HUMAnN2 following the developer’s instructions [37]. In each dataset, all predicted gene families and pathways were compared to those from metagenome sequencing in terms of their KEGG annotations that were downloaded from the KEGG website. For genes detected by both metagenome prediction tools and metagenome sequencing, we used two sets of methods to evaluate their consistency. In a first set of methods, we analyzed the Spearman correlation between predicted gene composition and those from metagenome sequencing. As a control, we permuted gene composition across samples 100 times and re-calculated Spearman correlation of gene composition between predictions and metagenome sequencing estimates.

In a second set of methods, we analyzed the consistency of metagenome prediction tools and metagenome sequencing in the *P* values they generated for null hypotheses of no association with metadata. For this purpose, *P* values were produced with a Wilcoxon test of the 2 distinguishable groups in each dataset (Table S1). *P* values from the Wilcoxon test were log10 transformed and multiplied by either 1 or − 1 to include the direction of change as indicated below:

$$ \mathrm{P}\_\mathrm{t}=\log 10\left(\mathrm{P}\right)\times \operatorname{sign}\left(\mathrm{mean}\_\mathrm{group}1-\mathrm{mean}\_\mathrm{group}2\right) $$

P_t is the transformed *P* value, P is the *P* value from Wilcoxon test, and the difference between means of the two distinguishable groups was used to add direction. We then estimated the consistency of the *P* values from metagenome prediction tools and metagenome sequencing with Spearman’s correlation. To determine whether this method is affected by the low variance of functional profiles, we permuted the metagenome sequencing produced gene compositions 100 times and re-calculated the *P* values and their correlation with the predictions. To correct for differences in sample size, each dataset was also subsampled to 5 samples per group to ensure that the different sample sizes of datasets were not unduly influencing our results. The predictions and metagenome sequencing were also compared in each of the 32 level 2 KEGG functional categories.

## Supplementary information

**Additional file 1.**

**Additional file 2.**

**Additional file 3.**

**Additional file 4.**

## Data Availability

The datasets analyzed in this study are publicly available with repositories and accession numbers listed in Table S1. R scripts used in this study are available at Github (https://github.com/ssun6/Inference_picrust). Additional requests and questions can be addressed to SS.
