# Supplementary material for: Inference-based accuracy of metagenome prediction tools varies across sample types and functional categories
Source: Microbiome. 2020 Apr 2;8:46. doi: 10.1186/s40168-020-00815-y (PMC7118876; doi:10.1186/s40168-020-00815-y)
Supplement: Supplementary file 2 — Additional file 1. [file 40168_2020_815_MOESM1_ESM.pdf]

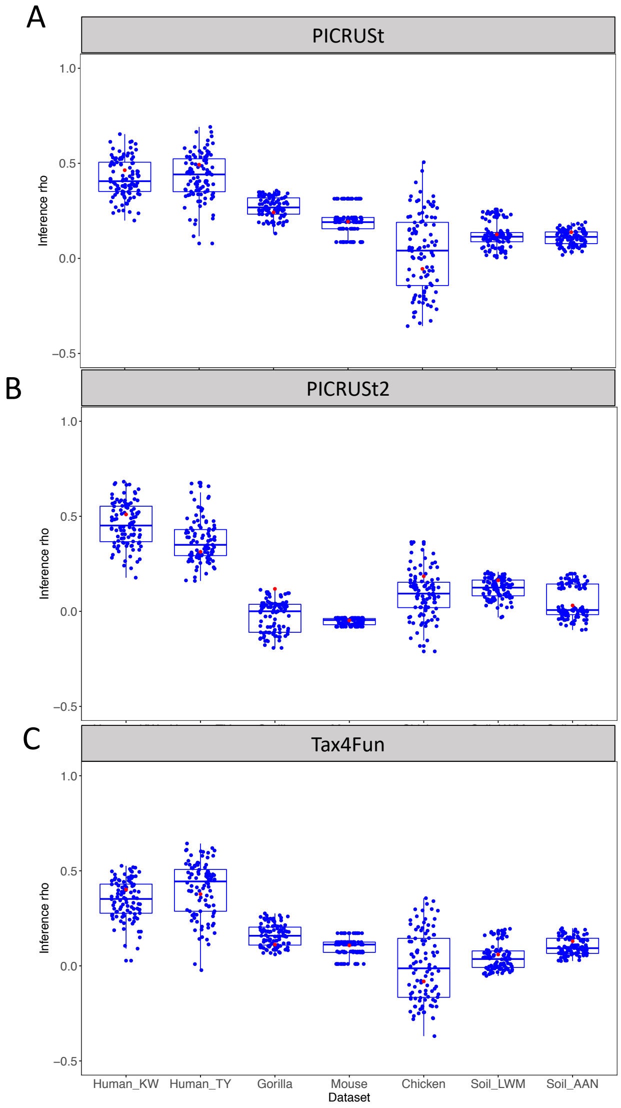

Fig. S1. The results of inference methods in whole and subsampled datasets. The red points are the inference correlations between metagenome prediction tools and unpermuted metagenome sequencing data for each dataset including all samples. The boxplots of blue points show the inference correlations between metagenome prediction tools and permuted metagenome sequencing data for each dataset that was subsampled to 5 samples per group.

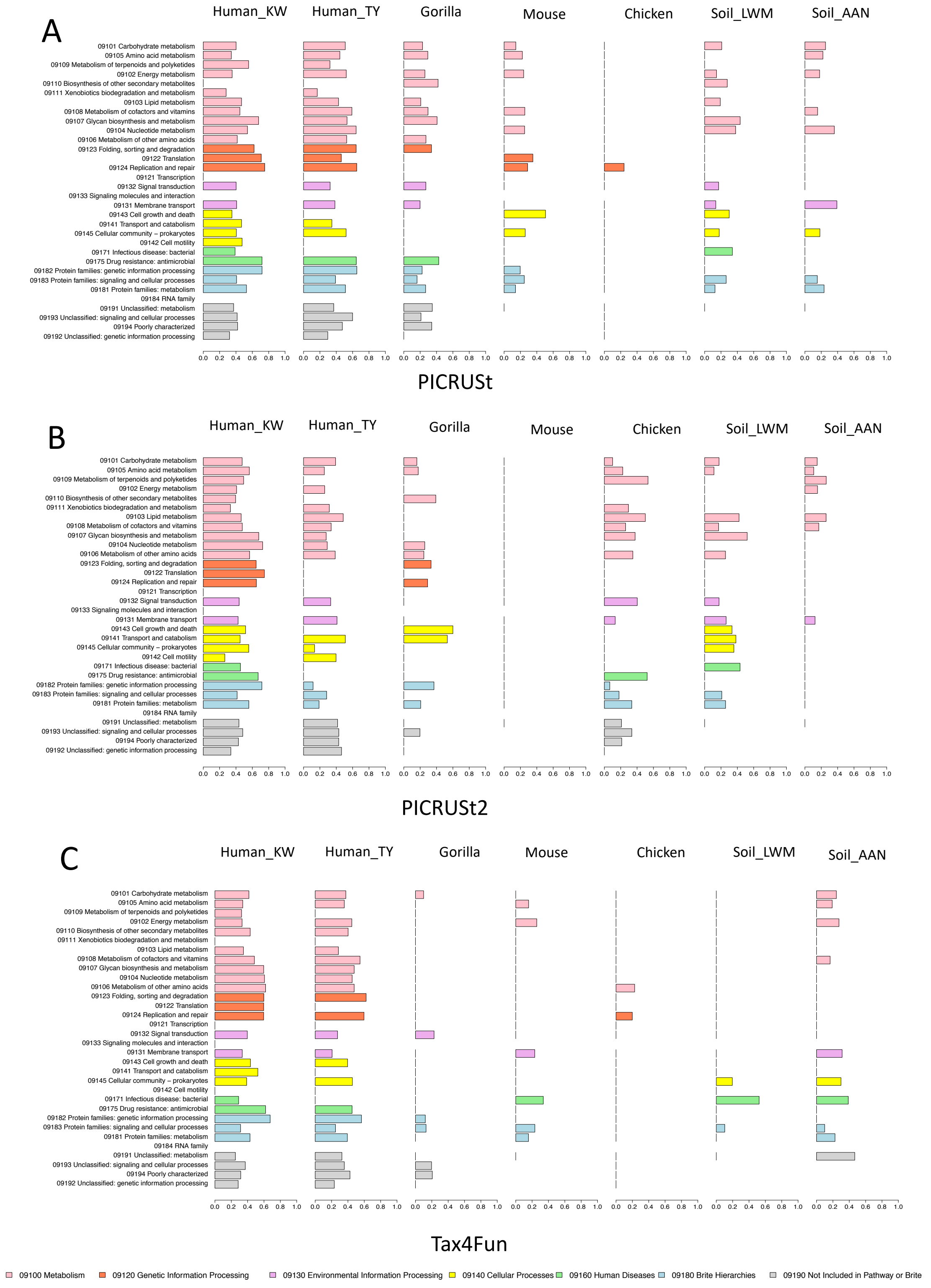

Fig. S2. Inference correlations (Spearman's rho) between metagenome sequencing and PICRUSt (A), PICRUSt2 (B) and Tax4Fun (C) in 32 KEGG functional categories at the second hierarchy level with the bar colors indicating the functional categories at the first hierarchy level. Negative and insignificant correlations are not shown.

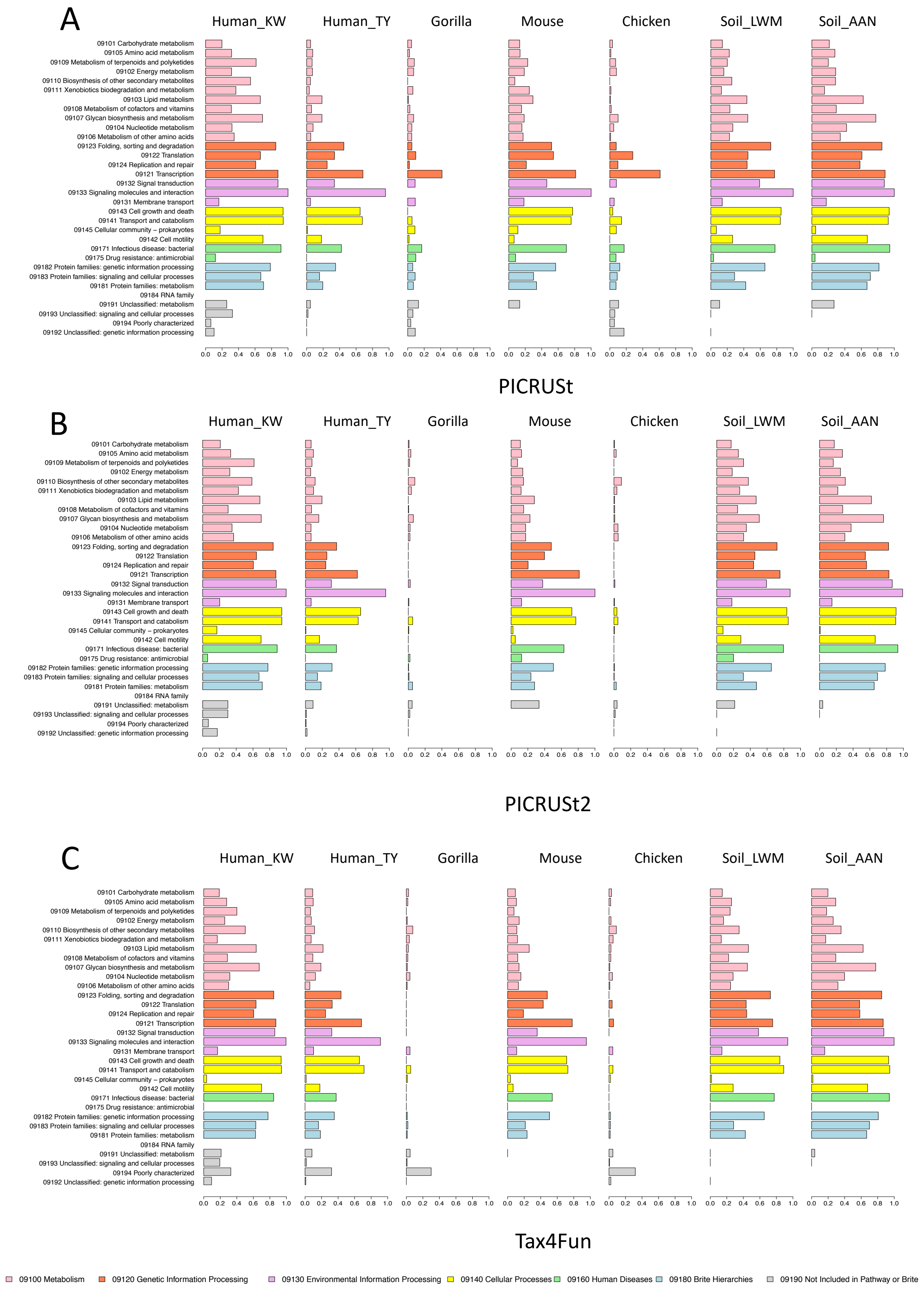

Fig. S3. The percentage of genes estimated with metagenome sequencing but not predicted by PICRUSt (A), PICRUSt2 (B) and Tax4Fun (C) in 32 KEGG functional categories at the second hierarchy level with the bar colors indicating the functional categories at the first hierarchy level.

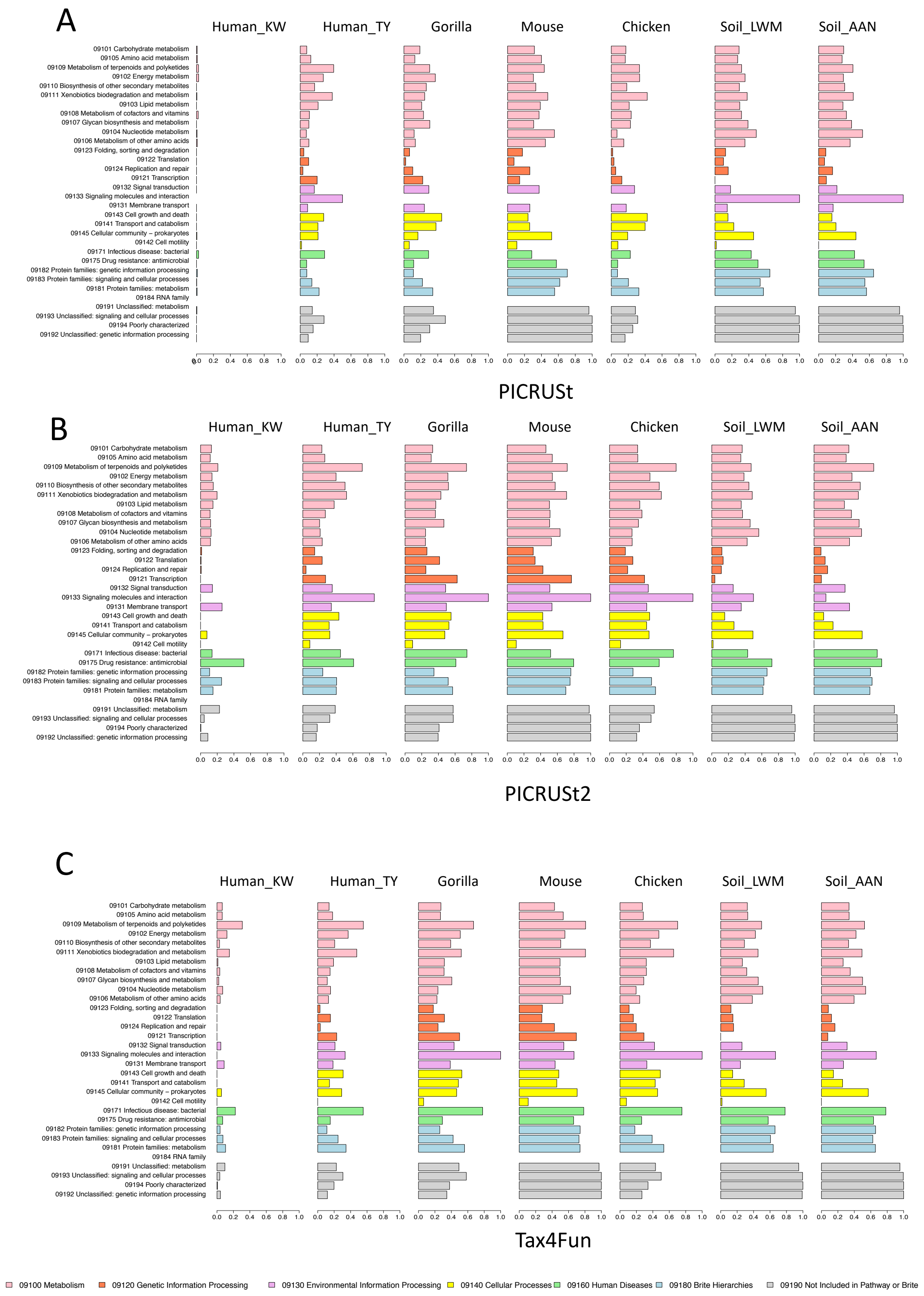

Fig. S4. The percentage of genes predicted by PICRUSt (A), PICRUSt2 (B) and Tax4Fun (C) but not showing in metagenome sequencing in 32 KEGG functional categories at the second hierarchy level with the bar colors indicating the functional categories at the first hierarchy level.
